# Supplementary material for: WordCommentsAnalyzer: A windows software tool for qualitative research
Source: F1000Res. 2018 Sep 4;7:536. Originally published 2018 May 3. [Version 2] doi: 10.12688/f1000research.14819.2 (PMC6137411; doi:10.12688/f1000research.14819.2)
Supplement: Supplementary file 3 [file f1000research-7-17614-s0002.tgz › b2a79496-4ada-47c4-9682-a99ce867836d.zip!/Eight Iranian Health Journals/Yafteh _24.docx]

© Yafteh  . This work is licensed under CC BY 4.0 (https://creativecommons.org/licenses/by/4.0/).

Volume & Issue: دوره 14 - شماره 4

Title: ارزیابی کیفیت بخش اورژانس بیمارستانهای دولتی خرمآباد براساس الگوی EFQM

http://yafte.lums.ac.ir/article-1-869-fa.html

مقدمه: بر اساس توصیه مرکز توسعه مدیریت و تحول اداری وزارت بهداشت، الگوی EFQM مناسب‌ترین مدل ارزیابی عملکرد برای پیاد‌ه‌سازی در بیمارستان‌های کشور است. در این پژوهش کیفیت بخش اورژانس بیمارستان‌های دولتی خرم‌آباد را براساس مدل فوق سنجیده ‌شده و با نتیجه ارزشیابی سالیانه وزارت بهدشت مقایسه شده است. بحث و نتیجه‌گیری: نتایج پژوهش بیانگر آن است که کیفیت بخش های اورژانس مورد مطالعه در مقایسه با استانداردهای مدل و نیز مطالعات مشابه به‌طور قابل ملاحظه‌ای پایین‌تر است؛ به نظر پژوهشگران، فاصله موجود با مطالعات خارجی منطقی و با مطالعات داخلی غیرمنطقی است. بخش‌های مورد مطالعه بیشترین امتیاز را در معیار فرایندها و کمترین آن را در معیار خط مشی و استراتژی کسب کردند. همچنین یافته‌ها بیانگر اختلاف زیاد بین نتایج ارزشیابی براساس مدل EFQM و نظام فعلی ارزشیابی وزارت بهداشت است، لذا پژوهشگران نظام کنونی ارزشیابی وزارت بهداشت را نیازمند بازنگری اساسی می‌دانند. یافته‌ها: بخش‌های اورژانس بیمارستان‌های تأمین اجتماعی، شهید مدنی، شهدای عشایر و خیریه عسلی از 1000 امتیاز مدل به ترتیب 216، 1/108، 9/129 و 1/72 امتیاز را کسب کردند. میانگین درصد امتیاز بخش‌های مورد مطالعه در معیارهای نه‌گانه EFQM به‌صورت زیر بود: رهبری 25/12%، خط مشی و استراتژی 1/7%، کارکنان 8/11%، مشارکت‌ها و منابع 8/13%، فرایندها 8/20%، نتایج مشتریان 3/8%، نتایج کارکنان 5/12%، نتایج جامعه 5/9% و نتایج کلیدی عملکرد 8/18% . مواد و روش‌ها: این مطالعه توصیفی- مقطعی در سال 1388 انجام شد. بخش اورژانس بیمارستان‌های دولتی خرم آباد جامعه آماری پژوهش بودند که همگی به روش سرشماری در نمونه پژوهش قرار گرفتند. ابزار گردآوری داده‌ها، پرسشنامه استاندارد مدل EFQM بود که با حضور پژوهشگران در بخش‌های مورد مطالعه تکمیل گردید. برای امتیازدهی از منطق RADAR و برای توافق‌نظر از فرایند دستیابی به اجماع EFQM استفاده شد؛ برای نمایش یافته‌ها از شاخص‌های آمار توصیفی، نرم افزار SPSS و نرم افزار EFQM استفاده شد.

Title: بررسی کمی و کیفی آلودگی بیوآئروسل‌های هوای بخش های مختلف بیمارستان‌های دولتی شهر همدان در سال 1390

http://yafte.lums.ac.ir/article-1-870-fa.html

مقدمه: با توجه به اینکه مطالعه کمی و کیفی آلودگی برروی بیوآئروسل‌های هوای بخش‌های مختلف بیمارستان‌های آموزشی دانشگاه علوم پزشکی همدان وجود نداشت، در این مطالعه تنوع و تراکم بیوآئروسل‌ها در هوای بیمارستان‌های شهر همدان به عنوان پایه‌ای برای مطالعات آتی و یا اقدامات بعدی بررسی شده است. بحث و نتیجه‌گیری: میزان آلودگی هوای برخی از بیمارستان‌ها به بیوآئروسل‌ها از نظر کمی بیشتر از استاندارد پیشنهادی بود. اکثر بیمارستان‌های مورد بررسی، فاقد سیستم های تصفیه هوا بودند و لذا بایستی در خصوص طراحی صحیح و نصب این تجهیزات اقدام شود. یافته‌ها: بیشترین و کمترین تراکم بیوآئروسل‌ها در بخش‌های زنان1 و اتاق عمل در بیمارستان زنان فاطمیه (cfu/m3 4/54 در مقابل cfu/m33/13) بدست آمد. مقایسه میزان میانگین تراکم کلی بیوآئروسل‌ها در بیمارستان‌ها با مقدار استاندارد پیشنهادی (cfu/m330) تفاوت معنی‌داری نشان نداد (3/0 = P). شایع‌ترین قارچ های جدا شده از هوای بیمارستان‌ها به ترتیب شامل پنی‌سیلیوم (06/32%)، کلادوسپوریوم (5/20%)، آسپرژیلوس فومیگاتوس (61/14%) و آسپرژیلوس نایجر (43/7%) بود. بیشترین درصد فراوانی باکتری‌های جدا شده استافیلوکوکوس کواگولاز منفی (49/32%)، باسیلوس‌ها (74/14%)، میکروکوک‌ها (68/13%)و استافیلوکوکوس اورئوس (34/11%) بودند. مواد و روش‌ها: در این پژوهش توصیفی ـ مقطعی، 30 بخش بیمارستانی از 5 بیمارستان آموزشی شهر همدان از نظر تنوع و تراکم بیوآئروسل‌های موجود در هوای آنها مورد بررسی قرار گرفت. تعداد بیش از 180 نمونه هوا در قسمت‌های مختلف با روش فیلتراسیون پیشنهادی از سوی کمیته بیوآئروسول (ACGIH) جمع آوری گردید. نمونه‌ها بلافاصله به محیط کشت آگار خونی و سابرودکستروز آگار منتقل و کشت داده شدند و سپس در آزمایشگاه تعداد و نوع کلنی‌های تشکیل شده، شمارش شده و تعیین گردیدند. در نهایت تراکم بیوآئروسول در هوا بر حسب cfu/m3 تعیین شد داده‌ها با نرم افزار آماری SPSS آنالیز گردید و تحلیل‌های آماری مربوطه انجام شد.

Title: بررسی اپیدمیولوژی بیماری سل در استان لرستان در سال‌های 88-1387

http://yafte.lums.ac.ir/article-1-871-fa.html

مقدمه: سل یکی از علل اصلی مرگ و میر در سراسر جهان شناخته می شود. 7/1 میلیارد نفر از مردم جهان به میکروب سل آلوده بوده و سالانه 8 میلیون مورد جدید سل و سه میلیون مرگ به دلیل این بیماری در کشورهای در حال توسعه رخ می دهد. بحث و نتیجه‌گیری: با توجه به شیوع بالای بیماری سل در استان و گزارش مواردی از شکست درمان و سل مقاوم به دارو، لزوم دقت بیشتر در تشخیص این بیماران و همچنین نظارت دقیق بر مصرف دارو توسط بیماران مشخص می گردد. یافته‌ها: در سال 1388، 198 مورد ابتلا به سل در استان شناسایی گردید.107 نفر مرد (54 درصد) و 91 نفر زن (46 درصد) بودند. 109(55%) نفر سل ریوی اسمیر مثبت ،32 (16%) نفر سل ریوی اسمیر منفی و 45 (7/22%) نفر سل خارج ریوی و 7 (5/3%) مورد عود سل داشتند. در سال 1387 نیز 149 مورد سل شناسایی شد که 79 نفر (54%) مرد و 70 نفر (46%) زن بودند. 68 (6/45%) نفر سل ریوی اسمیر مثبت و 35 (5/23%) نفر سل ریوی اسمیر منفی و 35 (5/23%) نفر سل خارج ریوی داشتند. مواد و روش‌ها: در این مطالعه پرونده افرادی که در سال 88 و 87 به مراکز بهداشتی درمانی استان مراجعه نموده بودند و نمونه‌های آنها از نظر باسیل اسید فاست مثبت بوده و یا دارای علائم منطبق با سل بود و توسط پزشک متخصص تحت درمان سل قرار گرفته بودند مورد بررسی قرار گرفت.

Title: خودمراقبتی و عوامل مرتبط با آن در بیماران دیابتی شهر خرم آباد

http://yafte.lums.ac.ir/article-1-873-fa.html

مقدمه: خودمراقبتی‌ فرایندی مهم در کنترل بیماری دیابت محسوب می‌شود. هدف این پژوهش ‌بررسی وضعیت خودمراقبتی و عوامل مؤثر بر آن در‌ بیماران مبتلا به دیابت ساکن شهر خرم آباد بود. بحث و نتیجه‌گیری: به منظور ارتقاء سطح سلامت بیماران دیابتی و کنترل مناسب بیماری‌ برنامه‌های آموزشی با هدف توانمندسازی بیماران در زمینه مراقبت از خود باید مورد توجه واقع شود. یافته‌ها: میانگین نمرات خودمراقبتی در بیماران 6/12±7/32 و وضعیت خودمراقبتی در حد متوسط بود. کمترین میانگین نمرات رفتارهای خودمراقبتی مربوط به انجام آزمایش قند‌خون (8/3±4/3)، انجام فعالیت فیزیکی (2/4±7/4) و مراقبت عمومی از پاها (9/4±9/4) بود. میانگین نمرات خودمراقبتی براساس میزان تحصیلات، محل سکونت، نوع‌ درمان ‌دریافتی و شبکه زندگی بیماران به صورت معناداری متفاوت بود (05/0(P<. مواد و روش‌ها: در این مطالعه مقطعی‌80 بیمار مبتلا به دیابت نوع 2 مراجعه کننده به درمانگاه‌های دیابت بیمارستان‌ها، پایگاه‌های‌ شهری و یک مطب فوق تخصصی غدد در سطح شهر خرم‌آباد به روش نمونه‌گیری در دسترس مورد بررسی قرار گرفتند. ابزار گردآوری اطلاعات مقیاس توبرت‌گلاسکو بود که وضعیت خودمراقبتی بیماران را در زمینه‌های مختلف طی هفت روز گذشته مورد ارزیابی قرار می‌داد. داده ها با استفاده از آزمون‌های تی مستقل و آنالیز واریانس یک‌طرفه تجزیه و تحلیل گردید.

Title: مقایسه کیفیت زندگی و سلامت عمومی بعد از زایمان سزارین و طبیعی در زنان مراجعه کننده به مراکز بهداشتی و درمانی شهر خرم آباد در سال 90-1388

http://yafte.lums.ac.ir/article-1-875-fa.html

مقدمه: این مطالعه با هدف مقایسه کیفیت زندگی و سلامت عمومی بعد از زایمان سزارین و طبیعی در زنان انجام شده است. مواد و روش‌ها: مطالعه حاضرمقایسه ای همگروهی است. جامعه پژوهش کلیه زنان باردار مراجعه کننده به مراکز بهداشتی درمانی شهر خرم آباد که با نمونه گیری هدفمند 380 نفر از آنان با داشتن شرایط ورود در مطالعه شرکت کردند. جمع آوری اطلاعات در طی 18 ماه در مرحله (ماه آخر بارداری و هشت هفته پس از زایمان) انجام شد. فرم‌های مصاحبه شامل اطلاعات دموگرافیک، پرسشنامه سلامت عمومی GHQ28 و پرسشنامه خلاصه شده کیفیت زندگی سازمان جهانی بهداشت بود. داده ها با آمار توصیفی و آزمون t آنالیز واریانس یک طرفه و تحلیل رگرسیون لجستیک، آزمون مجذور کای، من ویتنی در سطح معنی داری 5% بررسی شدند. بحث و نتیجه‌گیری: کیفیت زندگی در ابعاد مختلف آن و همچنین سلامت عمومی در مادران با زایمان طبیعی نمرات بهتر و مطلوب‌تری را نشان می‌دهد یافته‌ها: 250 نفر (146 نفر زایمان طبیعی و 104 نفر سزارین انتخابی) فرم‌های مصاحبه و پرسش نامه های پژوهشی را تکمیل کردند.. میانگین نمرات حیطه جسمی (001 /0 (P= و روانی )02/0 P=) کیفیت زندگی تفاوت معنی دار آماری نشان می‌دهد؛ و در حیطه محیط و اجتماعی و کل تفاوت معنی دار آماری دیده نمی‌شود. شانس ایجاد علائم مرضی در زنان با زایمان سزارین 26/3 برابر زنان زایمان طبیعی بوده است. شانس ایجاد علائم مرضی در مقیاس شکایت جسمانی در زنان با زایمان سزارین 09/3 برابر (001 /0 (P= افسردگی 75/1 برابر)02/0 P=)و اختلال کنش اجتماعی 68/1 برابر)04/0 P=) زنان زایمان طبیعی است .

Title: ارتباط بین سطح سرمی 25- هیدروکسی ویتامین D با شاخص های تن سنجی و برخی از پارامترهای بیوشیمیایی در زنان مبتلا به چاقی

http://yafte.lums.ac.ir/article-1-876-fa.html

مقدمه: مطالعات نشان داده اند که غلظت 25- هیدروکسی ویتامین D سرمی که بیانگر وضعیت ویتامین D بدن می باشد، ارتباط معکوسی با وضعیت چاقی داشته و خطر ابتلا به چاقی در افراد با سطوح بالای 25- هیدروکسی ویتامین D کمتر است. هدف از مطالعه حاضر بررسی ارتباط بین سطح سرمی 25- هیدروکسی ویتامین D با شاخص های تن سنجی، گلوکز ، اجزای لیپیدی ، انسولین سرمی و شاخص مقاومت انسولینی در زنان مبتلا به چاقی می باشد. بحث و نتیجه‌گیری: هیچ ارتباط معنی داری بین سطح سرمی 25- هیدروکسی ویتامین D با شاخص های تن سنجی (به استثنای وزن بدن)، گلوکز ، اجزای لیپیدی ، انسولین سرمی و شاخص مقاومت انسولینی در زنان چاق مورد مطالعه دیده نشد. یافته‌ها: میانگین سطح سرمی 25- هیدروکسی ویتامین D ، انسولین و همچنین مقاومت انسولینی بر اساس شاخص HOMA-IR در دو گروه چاق و کنترل بترتیب برابر با 66/16 ± 05/28 در مقابل 08/14 ± 72/25 ) 48/0 (p= ، 53/7 ± 97/25 در مقابل 32/9 ± 93/25) 98/0 (p= و 6/2 ± 39/6 در مقابل 5/2 ± 83/5 ) 30/0 (p= بود. از بین شاخص‌های تن سنجی ، 25- هیدروکسی ویتامین D تنها ارتباط معنی داری را با وزن افراد در گروه چاق داشت (326/0 , r= 033/0 p=) هیچ ارتباط معنی داری بین سطح سرمی 25- هیدروکسی ویتامین D با قند خون، اجزای لیپیدی، سطح انسولین سرمی و شاخص HOMA-IR در زنان چاق دیده نشد. مواد و روش‌ها: این مطالعه بر روی 43 زن 50- 20 سال مبتلا به چاقی و 43 زن سالم دارای وزن طبیعی صورت گرفت که از نظر سن و فعالیت بدنی با یکدیگر جور شده بودند. از تمامی شرکت کنندگان سه روز یادآمد غذایی 24 ساعته گرفته شد و اخذ نمونه خون به منظور اندازه‌گیری قند خون ، 25- هیدروکسی ویتامین D، اجزای لیپیدی و انسولین سرمی به عمل آمد.

Title: اثرمترونیدازول بر دنیتریفیکاسیون بیولوژیک سودوموناس استوتزری در فاضلاب

http://yafte.lums.ac.ir/article-1-877-fa.html

مقدمه: باکتری سودوموناس استوتزری از مهمترین و مؤثرترین باکتری‏‏های دنیتریفایر فاضلاب است. با توجه به اهمیت اثرات نیترات بر منابع آب و سلامتی انسان و نقش بازدارندگی مترونیدازول، این تحقیق با هدف بررسی اثر غلظت‏‏های مختلف مترونیدازول بر فعالیت دنیتریفایری سودوموناس استوتزری در فاضلاب انجام شد. مواد و روش‌ها: این مطالعه از نوع تجربی است. بعد از نمونه برداری فاضلاب خام، شناسایی و جداسازی باکتری سودوموناس‏استوتزری انجام شد. سپس سوسپانسیون باکتری در محیط کشت اختصاصی، غلظت ‏های مختلف مترونیدازول در آب مقطر و غلظت ‏های مختلف نیترات برای انجام آزمایشات حذف نیترات در شرایط انواکسیک آماده و آزمایشات بازدارندگی مترونیدازول بر فعالیت دنیتریفایری سودوموناس‏استوتزری در فاضلاب مصنوعی و واقعی انجام شد. یافته‌ها: مترونیدازول در غلظت ‏های 100 ،200 ، 300 و mg/l 400، در فاضلاب ‏های مصنوعی حاوی غلظت ‏های تا mg/l 550 نیترات، بر فعالیت دنیتریفایری سودوموناس استوتزری اثر بازدارندگی نداشت. غلظتmg/l 800 به بالای مترونیدازول در فاضلاب ‏های مصنوعی، و غلظتmg/l 500 به بالا در فاضلاب خانگی، اثر بازدارندگی داشتند. در غلظتmg/l 800 مترونیدازول، با افزایش غلظت سوبسترا ‏ (نیترات)، کاهش معنی‏دار سرعت حذف نیترات مشاهده شد. این باکتری به سه نمونه فاضلاب خانگی افزوده شد، ولی بر میزان حذف نیترات تأثیری نداشت. بحث و نتیجه‌گیری: با توجه به قابلیت حذف غلظت‌های بالای نیترات توسط باکتری سودوموناس استوتزری و مقاومت بالای آن در مقابل مترونیدازول، فرآیند دنیتریفیاسیون بیولوژیک، می‏تواند پتانسیل بالایی جهت کاربرد به منظور حذف غلظت‏‏های بالای نیترات از فاضلاب‏‏های صنعتی به ویژه فاضلاب صنایع دارویی داشته باشد.

Title: اثر 12 هفته تمرین ترکیبی (هوازی-مقاومتی) بر پروتئین واکنشگر- C (CRP) سرم و اینترلوکین-6 (IL-6) پلاسما در مردان غیر فعال

http://yafte.lums.ac.ir/article-1-878-fa.html

مقدمه: هدف این مطالعه بررسی تأثیر تمرین ترکیبی بر غلظت پروتئین واکنشگر- C (CRP)سرم و اینترلوکین-6 (IL-6) پلاسما در مردان غیر فعال بود. مواد و روش‌ها: در این تحقیق نیمه تجربی 20 مرد سالم (2±23 سال) داوطلب (بدون سابقه فعالیت ورزشی منظم، تغییر در وزن بدن بیشتر از 2 کیلوگرم، بیماری خاص و مصرف سیگار برای حداقل 6 ماه گذشته) با توده ی بدنی طبیعی (2±23 کیلوگرم بر متر مربع) بطور تصادفی در دو گروه (کنترل 10، تجربی 10) انتخاب شدند و فعالیت ترکیبی (تمرین هوازی شامل دویدن روی نوارگردان برای 20 دقیقه در 70-60 درصد حداکثر اکسیژن مصرفی (VO2 max) و تمرین مقاومتی با شدت 70 درصد یک تکرار بیشینه (RM1) با 10 تکرار در هر حرکت برای 2 ست) را به مدت 12 هفته (هر هفته سه جلسه) انجام دادند. بحث و نتیجه‌گیری: می توان گفت که اجرای تمرین های ترکیبی باعث کاهش غلظت CRP سرم در مردان غیر فعال می شود. احتمالا کاهش CRP سرم با بهبود درصد چربی بدن در این مردان همراه بود. پس فعالیت جسمانی بایستی بعنوان بخش اساسی در شیوه زندگی هر فرد بزرگسالی در نظر گرفته شود. یافته‌ها: نتایج نشان داد که سطوح CRP سرم، نسبت محیط کمر به لگن ( W/H) و درصد چربی بدن در گروه تمرینی به طور معناداری پایین تر از گروه کنترل بود (05/0P<). اما سطوح IL-6 پلاسما، BMI و وزن بدن بعد از برنامه فعالیت بدنی در دو گروه بدون تغییر باقی ماند. رابطه معنی داری در حالت پایه بین CRP با VO2 max (035/0=P و 67/0r=-) و درصد چربی بدن (018/0=P و 72/0r=) و همچنین بین BMI با درصد چربی بدن (045/0= P و 64/0r=) در کلیه آزمودنی ها مشاهده شد.

Title: بررسی شیوع مولکولی انگل بلاستوسیستیس هومینیس در مراجعه کنندگان به آزمایشگاه‌های شهر

http://yafte.lums.ac.ir/article-1-879-fa.html

مقدمه: بلاستوسیستیس هومینیس، انگلی تک یاخته ای، بی هوازی و زئونوز است که در روده بزرگ انسان و بسیاری از مهره داران دیگر یافت می شود . انتشاری جهانی و انتقال آن در میزبان های مختلف مستقیم و از طریق کیست به همراه آب و مواد غذایی آلوده صورت می گیرد و عواملی مانند فرهنگ بهداشتی، فصول سال، ارتباط باحیوانات و سن در شیوع آن مؤثرند. برخلاف گذشته، بسیاری از مطالعات دهه اخیر،‌ بیماریزایی بالقوه آن را ت‍‍أیید و علائم گوارشی و خارج گوارشی متعددی را به آن نسبت می‌دهند. این انگل دارای مرفولوژی، سیر تکاملی و تکثیر عجیب و منحصر به فردی است. این مطالعه با هدف بررسی شیوع مولکولی این انگل در مراجعه کنندگان به آزمایشگاه‌های شهر خرم‌آباد انجام شد. مواد و روش‌ها: در این مطالعه که برای اولین بار در استان لرستان انجام گرفت، 511 نمونه مدفوع از افراد مراجعه کننده به آزمایشگاه های شهر خرم آباد جمع آوری گردید که پس از استخراج DNA با روش PCR ، جهت تشخیص مولکولی انگل بلاستوسیستیس مورد مطالعه و بررسی قرارگرفتند. یافته‌ها: از511 نمونه که مورد بررسی قرار گرفته ،33 نمونه (5/6%) به بلاستوسیستیس آلوده بودند. بحث و نتیجه‌گیری: شاخص‌های مرفولوژیک انگل و سایر عوامل مداخله گر، تشخیص میکروسکوپی آن را با چالش‌هایی مواجه ساخته‌اند و روش تشخیصی PCR که حساسیت و ویژگی بالاتری نسبت به سایر روش های تشخیصی دارد، توصیه می شود. محققان، دنیا را بر حسب میزان شیوع انگل بلاستوسیستیس به دو گروه توسعه یافته تا 10% و در حال توسعه تا 50% تقسیم نموده اند که شیوع 5/6% جمعیت مذکور در شهر خرم آباد،‌ در محدوده کشورهای گروه اول است که با توجه به منبع آب آشامیدنی شهر خرم آباد که اکثراً از چشمه ها تأمین می‌شود، قابل تفسیر است ولی از آنجا که دامداری یکی از مشاغل مهم استان محسوب می شود،‌ رعایت نکات بهداشتی و پوشش مناسب را هنگام برخورد با حیوانات ضروری می نماید. ضمناً نتایج این تحقیق و مطالعات مشابه در کشور های در حال توسعه از یک طرف و شیوع در حال افزایش 23درصدی‌ در ایالات متحده آمریکا به عنوان یک کشور توسعه یافته از طرف دیگر،‌ معادله تقسیم بندی قبلی را به هم زده است.

Title: بررسی فراوانی ناهنجاری‌های ستون فقرات و عوامل مرتبط با آن در دانشجویان

http://yafte.lums.ac.ir/article-1-880-fa.html

مقدمه: در هر منطقه جغرافیایی با توجه به فرهنگ اجتماعی و اقلیمی، ناهنجاری‌ها و عوارض خاصی ممکن است در ستون فقرات رخ دهند که در صورت شناسایی، اغلب با حرکات و الگوهای رفتاری خاص می‌توانند اصلاح گردند. بدین منظور، پژوهش حاضر با هدف بررسی میزان و علل ناهنجاری‌های ستون فقرات دانشجویان دانشگاه آزاد اسلامی واحد خرم‌آباد انجام پذیرفت. مواد و روش‌ها: مطالعه توصیفی- تحلیلی حاضر، در سال های 1390-1389 بر روی دانشجویان دانشگاه آزاد اسلامی خرم‌آباد انجام گرفت. از میان 14000 نفر دانشجو، تعداد 500 نفر (250 نفر زن و 250 نفر مرد) به طور خوشه ای چند مرحله ای از میان دانشجویانی که در نیمسال دوم سال تحصیلی 90-89 مشغول به تحصیل بودند، مورد بررسی قرار گرفتند. ابتدا در طی پرسشنامه‌ا‌ی مشخصات فردی دانشجویان، سابقه بیماری و عادات روزمره آنان پرسیده شد. سپس وزن و قد دانشجویان و ناهنجاری‌های ستون فقرات آنان بررسی گردید. برای اندازه گیری ناهنجاری‌های ستون فقرات از آزمون استاندارد نیویورک و صفحه شطرنجی استفاده شد. همچنین جهت اندازه‌گیری زوایای قوس مهره‌های کمری و پشتی از خط کش منعطف استفاده گردید. بحث و نتیجه‌گیری: تحقیق حاضر نشان دهنده آمار بالای شیوع ناهنجاری های ستون فقرات در دانشجویان دانشگاه آزاد اسلامی واحد خرم آباد است که عواملی چون فقر حرکتی، عادات نادرست در راه رفتن، ایستادن، نشستن، خوابیدن، حمل نادرست کیف و وسایل، نقش بسزایی در ایجاد هر یک از این ناهنجاری ها داشته اند. یافته‌ها: 2/83% (208 نفر) مردان و 2/87% (218 نفر) زنان دانشجو دارای حداقل یک عارضه بودند. بیشترین ناهنجاری در مردان مربوط به عارضه کیفوز (6/83%) و در زنان مربوط به عارضه شانه نابرابر (76%) بود. عارضه افتادگی شکم (4/10%) در مردان و عارضه انحراف بالاتنه (2/19%) در زنان، کمترین میزان ناهنجاری را نیز به خود اختصاص دادند. همچنین میان عادات غلط خوابیدن و مطالعه با لوردوز و کیفوز، میان عادات نادرست حمل کیف و وسایل و شانه نابرابر، عادات غلط مطالعه و سر به جلو و عادات غلط نشستن در خانه و کیفوز ارتباط معنی‌داری مشاهده شد (05/0P<).

Title: مقایسه یافته‌های آزمایشگاهی در بیماران تحت جراحی بای‌پس عروق کرونر با و بدون استفاده از پمپ قلبی- ریوی

http://yafte.lums.ac.ir/article-1-881-fa.html

مقدمه: تلاش‌ها برای انجام جراحی عروق کرونر به روشی که عوارض کمتری داشته باشد، منجر به انجام مطالعات زیادی شده است. این مطالعات دو روش جراحی عروق کرونر، با استفاده از پمپ قلبی-ریوی و بدون استفاده از پمپ قلبی-ریوی را با هم مقایسه کرده اند. لذا این مطالعه به منظورمقایسه یافته های آزمایشگاهی دراین دو گروه از بیماران انجام شده است. بحث و نتیجه‌گیری: با توجه به وقوع کمتر آسیب حاد کلیوی و سطح پایین تر قند خون و کراتینین سرم و عوارض احتمالی ناشی از این موارد، روش بدون استفاده از پمپ قلبی-ریوی می‌تواند در بیماران روش مطلوب تری برای درمان باشد. با این حال در انتخاب روش جراحی به سایر مؤلفه‌ها نیز باید توجه داشت. یافته‌ها: وقوع آسیب حاد کلیوی و افزایش میزان کراتینین سرم، نیتروژن اوره خون، پتاسیم سرم و سطح گلوکز خون در گروه استفاده از پمپ قلبی-ریوی بیشتر بود (05/0< P). اما مقادیر سدیم سرم در دو گروه تفاوت معناداری نداشت (84/0=P). مواد و روش‌ها: در یک مطالعه گذشته نگر، 167 بیمار تحت درمان با جراحی عروق کرونر به دو روش با و بدون استفاده از پمپ قلبی-ریوی با استفاده از روش نمونه گیری تصادفی سیستماتیک از لیست بیماران بیمارستان قلب جماران (تهران) در سال1387 انتخاب شدند و از نظر مقادیر قند خون، کراتینین، سدیم و پتاسیم سرم و وقوع آسیب حاد کلیه با هم مقایسه شدند.
